# Professor Hermann Nothnagel

**Published:** 1905-08

**Authors:** 


Professor Hermann Nothnagel died at Vienna, July 7, 1905, aged 63 years. Professor Nothnagel was a distinguished clinician, a celebrated teacher, and an author of many valuable works on medicine. His latest work, Nothnagel's Encyclopedia of Practical Medicine, is now going through the press.

